# Elevated α‐Synuclein Aggregate Levels in the Urine of Patients with Isolated REM Sleep Behavior Disorder and Parkinson's Disease

**DOI:** 10.1002/ana.27250

**Published:** 2025-04-26

**Authors:** Laura Müller, Pelin Özdüzenciler, Charlotte Schedlich‐Teufer, Aline Seger, Hannah Jergas, Gereon R. Fink, Dieter Willbold, Michael Sommerauer, Michael T. Barbe, Gültekin Tamgüney

**Affiliations:** ^1^ Mathematisch‐Naturwissenschaftliche Fakultät, Institut für Physikalische Biologie Heinrich‐Heine‐Universität Düsseldorf Düsseldorf Germany; ^2^ Institute of Biological Information Processing (Structural Biochemistry: IBI‐7) Forschungszentrum Jülich Jülich Germany; ^3^ Department of Neurology, Faculty of Medicine and University Hospital Cologne University of Cologne Köln Germany; ^4^ Cognitive Neuroscience Institute of Neuroscience and Medicine (INM‐3), Forschungszentrum Jülich Jülich Germany; ^5^ Center of Neurology, Department of Parkinson's, Sleep and Movement Disorders University Hospital Bonn Bonn Germany

## Abstract

Parkinson's disease (PD) is a neurodegenerative disorder characterized by α‐synuclein aggregation in neurons. Recent advances suggest α‐synuclein aggregates could serve as a biomarker for PD and related synucleinopathies. This study used surface‐based fluorescence intensity distribution analysis (sFIDA) to measure α‐synuclein aggregates in urine. Patients with PD and isolated rapid eye movement sleep behavior disorder, a precursor to PD, had elevated concentrations compared with healthy controls. Sensitivity and specificity were 83% and 65% for PD versus controls and 89% and 62% for isolated rapid eye movement sleep behavior disorder versus controls. The findings highlight sFIDA's potential for diagnosing synucleinopathies. ANN NEUROL 2025;98:147–151

Parkinson's disease (PD) is a progressive neurodegenerative disease characterized by the pathological aggregation of α‐synuclein in neurons.^1^, ^2^ The diagnosis of PD predominantly depends on clinical evaluation and brain imaging.^3^ Efforts to identify clinical and biological markers of disease heterogeneity and progression in PD are ongoing, yet a quantitative biomarker remains unavailable.^4^ An emerging biomarker for PD is aggregated α‐synuclein, found in cerebrospinal fluid (CSF) and other biosamples.^5^ Importantly, patients with isolated rapid eye movement sleep behavior disorder (iRBD), a prodrome of PD and related synucleinopathies, also accumulate pathological α‐synuclein aggregates in the nervous system.^6^

Quantifying α‐synuclein aggregates in biosamples is challenging due to low concentrations and excess α‐synuclein monomers.^7^ To address these challenges, we recently adapted surface‐based fluorescence intensity distribution analysis (sFIDA) to measure α‐synuclein aggregates in CSF and stool and demonstrated that sFIDA is a highly sensitive assay for quantifying α‐synuclein aggregates in biosamples.^8^, ^9^ The sFIDA utilizes the Syn211 antibody, which specifically targets amino acids 121 to 125 of α‐synuclein to capture α‐synuclein species. Because the Syn211 antibody recognizes only a single linear epitope on α‐synuclein, its use as the detection antibody ensures precise quantification of aggregated α‐synuclein species exclusively, as monomers with a single linear epitope are captured but remain undetected. Labeling the detection antibody with fluorophores and imaging the surface using confocal microscopy achieves near single particle sensitivity.

Considering that cellular components from the central and peripheral nervous systems can be released and distributed throughout the body and bloodstream, we hypothesized that aggregated α‐synuclein species might likewise be detectable in the urine of patients with PD and iRBD.^4^, ^8^, ^9^, ^10^ The aim of this study was to detect and quantify α‐synuclein aggregates in urine and, more importantly, to demonstrate their potential application in the diagnosis of patients with synucleinopathies.

## Methods

### Participants

Patients with PD and iRBD and healthy controls (HCs) were enrolled at the Department of Neurology of the University Hospital Cologne and diagnosed based on established criteria, including polysomnography for patients with iRBD.^9^, ^11^ Urine samples were collected between July 2020 and September 2021 and stored at −80°C. All patient data and samples were pseudonymized. The study was approved by the research ethics committees of the participating institutions. Participants provided written informed consent.

### Sample Preparation

Urine samples were thawed at 4°C for 30 minutes, transferred to tubes coated with 3% bovine serum albumin (BSA; Applichem), and adjusted to 3% BSA and 1× Halt protease inhibitor cocktail (Thermo Fisher Scientific). After centrifugation at 4000 × g for 30 minutes at 4°C, the supernatants were transferred to BSA‐coated centrifugal concentrators (Sartorius) with a 10 kDa cutoff and concentrated 10‐fold by centrifugation at 12,000 × g for 15 to 30 minutes at 4°C. Concentrated samples were transferred to BSA‐coated tubes and stored at −80°C.

### sFIDA Protocol

To capture α‐synuclein aggregates, a 384‐well glass‐bottom microplate (Thermo Fisher Scientific) was incubated with 7.5 μg/ml of the Syn211 antibody (Santa Cruz Biotechnology) in 1× phosphate‐buffered saline overnight at 4°C. Subsequently, the plate was washed 5 times with 80 μl Tris‐buffered saline (TBS) with 0.05% Tween 20 (TBS‐T), and then 5 times with TBS. To block free binding sites, the wells were incubated with 0.5% fat‐free dried milk powder in TBS with 0.03% ProClin (Sigma Aldrich, TBS‐ProClin) for 3 hours at room temperature. Next, the wells underwent 5 washes with 80 μl of TBS‐T and 5 washes with TBS. The α‐synuclein‐coated silica nanoparticles (SiNaPs), which functioned as a standard for protein quantification, were prepared as previously described^8^, ^9^, ^12^ and diluted with 0.1% BSA and 0.05% Tween 20 in TBS‐ProClin. Four replicates of 20 μl of each SiNaP dilution or 20 μl of the 10× concentrated urine samples were added to the wells and incubated for 1 hour at room temperature. Subsequently, the plate was washed 5 times with TBS. To detect α‐synuclein aggregates, the wells were incubated with the fluorescent Syn211‐CF633 and Syn211‐CF488A antibodies,^9^ each at 0.625 μg/ml, in TBS‐T containing 0.1% BSA. Finally, the plate was washed 5 times with TBS and the buffer exchanged to TBS‐ProClin for measurement. An automated microtiter plate washer (405 LS Microplate Washer, BioTek) carried out all washing steps.

### Image‐Data Acquisition and Analysis

Images were captured using an IN Cell Analyzer 6500HS (GE Healthcare) with a 40× objective through 2‐channel confocal fluorescence imaging. A total of 25 images per well, each measuring 2,040 × 2,040 pixels, were captured for each channel. The sFIDAta version 2.2.7 was utilized for the automated detection and elimination of images with artifacts, and for quantifying colocalized pixels in both channels (sFIDA readout).^9^ To minimize background, a cutoff was established as the gray value at which 0.01% of all pixels in images of the buffer control were positive. Only pixels with gray values above the cutoff were analyzed. The limit of detection (LOD) was defined as the sFIDA readout of the buffer control plus 3 standard deviations. A calibration curve was fitted using sFIDA readouts of dilutions of SiNaPs of known concentration and second order polynomial regression. This calibration curve was used to calculate a concentration for each sFIDA readout.

## Results

### Descriptive Analysis of the Patient and Control Groups

We collected urine samples of 93 patients with PD and 72 patients with iRBD and 52 HCs. Demographic, clinical, and statistical information for these cohorts is available in the Table 1 and Supplementary Table S1 and Supplementary Figure S1. There was a gender bias toward men in the patient cohorts, and a slight bias toward women in the control group. HCs were, on average, 10 to 12 years younger than the patients. We did not observe significant differences in the duration of education. The cognitive performance (DemTect score) of the HCs was significantly higher than that of the patients.^13^ The patients with PD scored significantly higher than patients with iRBD on the Movement Disorder Society's Unified Parkinson's Disease Rating Scale Part III.^14^ On average, patients with PD scored 3.0 ± 0.9 on the Hoehn and Yahr scale and improved 42 ± 19% on the levodopa challenge test.^15^ The patients with PD scored significantly higher on the Non‐Motor Symptoms Scale for PD than the patients with iRBD or the HCs.^16^ Based on the Cleveland Clinic Constipation Scoring System, patients with PD were not more constipated than the patients with iRBD and the HCs.^17^ The patients with PD also suffered more from hyposmia, reflected in a significantly lower olfactory testing score, than the patients with iRBD or the HCs. In addition, patients with PD scored significantly higher in the screening questionnaire for parkinsonism compared with patients with iRBD and the HCs.^18^ The results of the RBD screening questionnaire also showed significant differences between patients with iRBD and patients with PD or the HCs, as well as between patients with PD and the HCs.^19^

**TABLE 1:** Demographic and Clinical Information on Patients and Controls That Donated Urine Samples

	Group			*p* Value		
	PD	iRBD	HC	PD vs. HC	PD vs. iRBD	iRBD vs. HC
Number	93	72	52	N/A	N/A	N/A
Female, number (percentage)	29 (31.2)	10 (13.9)	31 (59.6)	*p* < 0.001	*p* < 0.01	*p* < 0.0001
Age, yr ± SD	64.3 ± 9.7	66.3 ± 6.4	54.8 ± 16.8	*p* < 0.01	n.s.	*p* < 0.001
Education, yr ± SD	15.4 ± 4.1	15.9 ± 4.0	16.0 ± 3.3	n.s.	n.s.	n.s.
Disease duration, yr ± SD	9.0 ± 5.8	7.4 ± 5.4	N/A	N/A	n.s.	N/A
DemTect, score ± SD	14.0 ± 3.1	14.8 ± 2.3	16.1 ± 2.0	*p* < 0.0001	n.s.	*p* < 0.01
MDS‐UPDRS III, score ± SD	23.7 ± 14.6	4.0 ± 2.7	N/A	N/A	*p* < 0.0001	N/A
Non‐Motor Symptoms Scale, score ± SD	30.3 ± 22.8	16.2 ± 18.0	11.0 ± 11.0	*p* < 0.0001	*p* < 0.0001	n.s.
CCCSS, score ± SD	4.1 ± 4.0	2.8 ± 2.7	2.4 ± 2.5	n.s.	n.s.	n.s.
Olfactory testing, score ± SD	5.4 ± 2.5	6.6 ± 2.7	10.3 ± 1.8	*p* < 0.0001	*p* < 0.05	*p* < 0.0001
Screening questionnaire for parkinsonism, score ± SD	5.6 ± 2.2	0.3 ± 0.8	0.3 ± 0.7	*p* < 0.0001	*p* < 0.0001	n.s.
RBD screening questionnaire, score ± SD	4.9 ± 3.0	9.0 ± 2.7	2.1 ± 2.2	*p* < 0.0001	*p* < 0.0001	*p* < 0.0001
Hoehn and Yahr, score ± SD	3.0 ± 0.9	N/A	N/A	N/A	N/A	N/A
Improvement of MDS‐UPDRS III on Levodopa challenge test, % ± SD	42 ± 19	N/A	N/A	N/A	N/A	N/A

CCCSS = Cleveland Clinic Constipation Scoring System; HC = healthy control; iRBD = isolated rapid eye movement sleep behavior disorder; MDS‐UPDRS III = Movement Disorder Society's Unified Parkinson's Disease Rating Scale Part III; n.s. = not significant; N/A = not available; PD = Parkinson's disease; RBD = rapid eye movement sleep behavior disorder; SD = standard deviation.

### sFIDA Measurements of α‐Synuclein‐Coated Silica Nanoparticles

**FIGURE 1:**
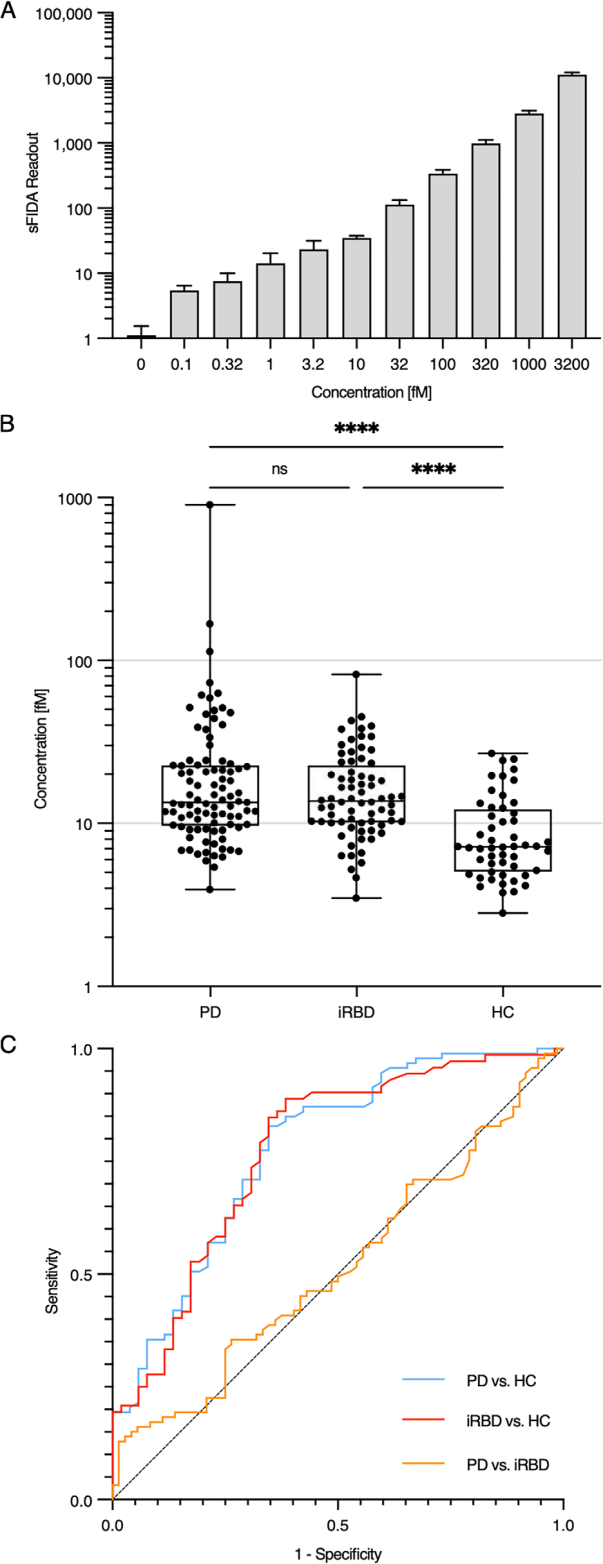
Concentration of α‐synuclein aggregates in urine samples and receiver operating characteristics (ROC) curves. (A) sFIDA measurements of dilutions of α‐synuclein‐coated silica nanoparticles (SiNaPs) were utilized as a standard for signal calibration. (B) The median concentration of α‐synuclein aggregates in 10× concentrated urine of patients with Parkinson's disease (PD; 13.5 fM) and isolated rapid eye movement sleep behavior disorder (iRBD; 13.7 fM) was significantly (****p < 0.0001) higher than in the urine of the healthy controls (HCs; 7.2 fM). The sFIDA readouts were converted to concentrations using sFIDAta version 2.2.7. Significance was determined using GraphPad Prism version 10.4.0 and the Kruskal‐Wallis test (ns = non‐significant). (C) A comparison of patients with PD with healthy controls revealed a sensitivity of 83% at a specificity of 65% with an area under the curve (AUC) of 0.7763 (*p* < 0.0001). A comparison between patients with iRBD and healthy controls yielded a sensitivity of 89% and a specificity of 62% with an AUC of 0.7743 (*p* < 0.0001). The ROC analyses were performed using GraphPad Prism version 10.4.0. The optimal combination of sensitivity and specificity was calculated with a maximized Youden's index.

To establish a standard for signal calibration, we utilized dilutions of α‐synuclein‐coated silica nanoparticles (SiNaPs) ranging from 0.1 to 3,200 fM (Fig 1A).^12^ The mean intra‐assay coefficient of variation (CV%) was determined based on the sFIDA readout of 4 replicates of each dilution of the SiNaPs standard, yielding a value of 19.9% (Supplementary Table S2). The sFIDA readout of 3,200 fM SiNaPs without a capture antibody was reduced by 99.7%. Based on the buffer control, the LOD was determined to be 0.74 fM.

### α‐Synuclein Aggregate Concentrations Are Elevated in the Urine of patients with PD and patients with iRBD

We used sFIDA to measure α‐synuclein aggregate concentrations in the urine of HCs and patients with PD and iRBD. Urea and other urine components could potentially confound sFIDA performance. However, urea concentrations between 100 and 800 mM did not affect the sFIDA readout (Supplementary Fig S2). We used SiNaPs to calibrate the sFIDA readouts and calculate the concentrations of α‐synuclein aggregates (Fig 1B, Supplementary Table S1). The median concentration of α‐synuclein aggregates in 10× concentrated urine samples from patients with PD (13.5 fM) and patients with iRBD (13.7 fM) was significantly elevated (*p* < 0.0001) compared with those from the HCs (7.2 fM) using the Kruskal‐Wallis test. Including only HCs over 50 years old, with a median age of 64.4 ± 7.6 years, closely matched the median ages of the PD (64.3 ± 9.7 years) and iRBD (66.3 ± 6.4 years) cohorts while preserving the statistically significant differences observed in urinary α‐synuclein fibril concentrations. We observed no significant difference between patients with PD and patients with iRBD. The receiver operating characteristic (ROC) curves (Fig 1C) revealed an 83% sensitivity and 65% specificity for patients with PD versus the HCs, and an 89% sensitivity and 62% specificity for patients with iRBD versus the HCs. We observed no correlation between α‐synuclein aggregate concentrations in urine and other disease‐relevant scores (Supplementary Table S3).

## Discussion

We demonstrated that α‐synuclein aggregate concentrations are significantly (*p* < 0.0001) elevated in the urine of patients with PD and patients with iRBD compared to the HCs. This study provides the first detection of urinary α‐synuclein aggregates in patients with iRBD that typically progress to defined parkinsonism within a few years.^20^ Notably, the concentrations in patients with iRBD matched those we observed in patients with PD, possibly due to an initiation of α‐synuclein pathology in the peripheral nervous system in patients with iRBD.^21^ These findings corroborate and expand on a previous study reporting higher (*p* < 0.05) urinary α‐synuclein aggregate concentrations in 21 patients with PD compared with 11 HCs.^22^ Whereas sFIDA for urine is nearly as sensitive as the seed amplification assay for detecting α‐synuclein aggregates in CSF samples of various synucleinopathies, it is less specific.^4^ We also observed α‐synuclein aggregates in the urine of some control subjects, possibly because sFIDA cannot differentiate between seeding‐competent aggregates, which are neurotoxic, and alternate seeding‐incompetent forms that may be present in healthy individuals.^23^ Alternatively, some control subjects may be in the clinical prodromal stage of PD. The origin of urinary α‐synuclein aggregates remains uncertain but may be linked to blood or peripheral nerves.^5^ In summary, our results demonstrate that pathological α‐synuclein aggregates are excreted in urine and can be quantified using sFIDA, highlighting its potential for diagnosing synucleinopathies in their early stages.

## Author Contributions

M.S., M.T.B., G.R.F., D.W., and G.T. contributed to the conception and design of the study; C.S.T., A.S., H.J., L.M., P.Ö., M.S., M.T.B., and G.T. contributed to the acquisition and analysis of data; L.M., G.R.F., D.W., M.S., M.T.B., and G.T. contributed to drafting the text or preparing the figures.

## Potential Conflicts of Interest

D.W. is co‐founder of attyloid GmbH, which is commercializing the sFIDA assay. All other authors have nothing to report.

## Supporting information


**Data S1** Supporting Information.

## Data Availability

The authors confirm that the data supporting the findings of this study are available within the article and its supplementary materials.
