# Supplementary material for: Elevated α‐Synuclein Aggregate Levels in the Urine of Patients with Isolated REM Sleep Behavior Disorder and Parkinson's Disease
Source: Ann Neurol. 2025 Apr 26;98(1):147–51. doi: 10.1002/ana.27250 (PMC12174725; doi:10.1002/ana.27250)
Supplement: Supplementary file 1 — Data S1 Supporting Information. [file ANA-98-147-s001.pdf]

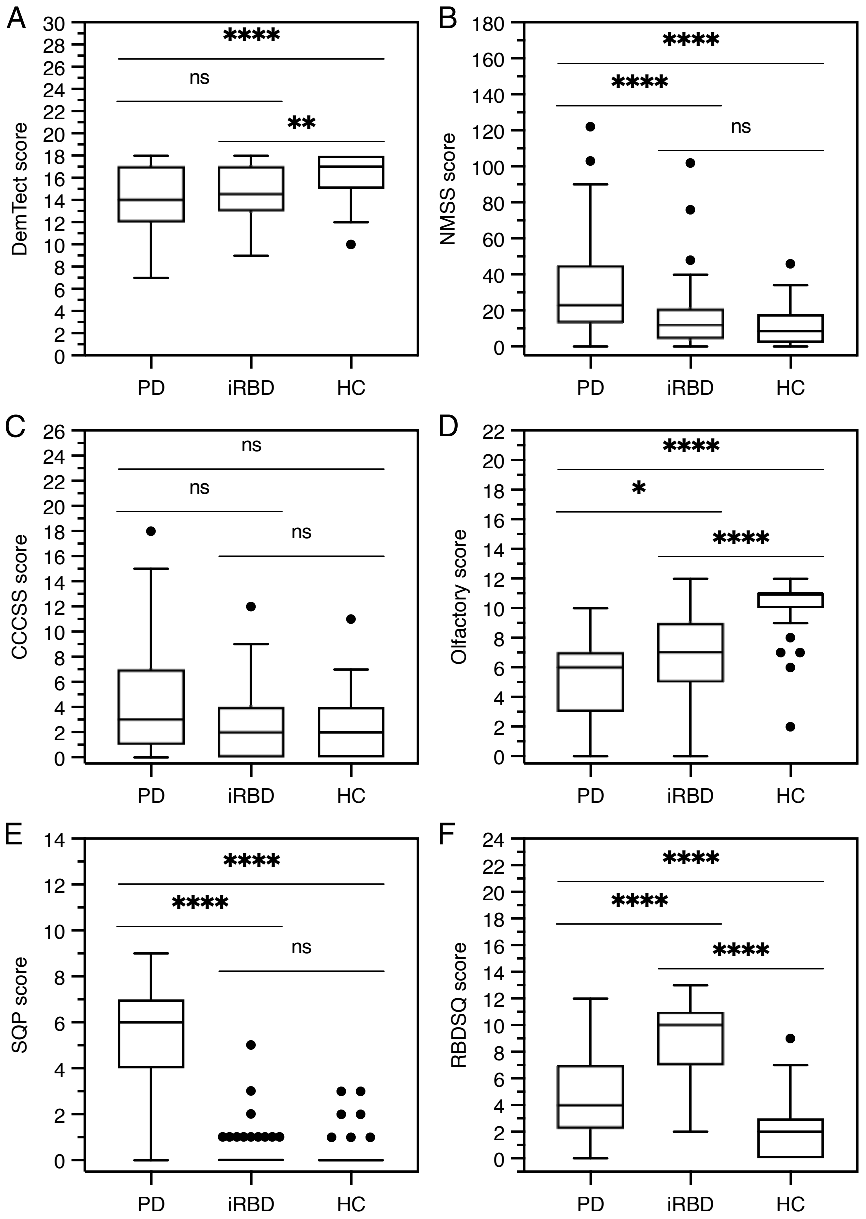

Figure S1: Characteristics for PD and iRBD patients and healthy controls based on their performance in various tests and screening questionnaires. (A) The DemTect score showed that the cognitive performance of healthy controls (16.1 ± 2.0) was significantly higher ($p < 0.0001$) than that of PD (14.0 ± 3.3) and iRBD patients (14.8 ± 2.3, $p < 0.01$). (B) Based on the Non-Motor Symptoms Scale (NMSS), the performance of PD patients (30.3 ± 22.8) was significantly worse ($p < 0.0001$) than that of iRBD patients (16.2 ± 18.0) and healthy controls (11.0 ± 11.0). (C) Based on the Cleveland Clinic Constipation Scoring System (CCCSS), there was no significant difference in constipation of PD patients (4.1 ± 4.0), iRBD patients (2.8 ± 2.7), and healthy controls (2.5 ± 2.5). (D) The olfactory performance of PD patients (5.4 ± 2.5) was significantly worse ($p < 0.05$) than that of iRBD patients (6.6 ± 2.7). That of PD and iRBD patients was significantly worse ($p < 0.0001$) than that of healthy controls (10.3 ± 1.8), based on correctly identified Sniffin' sticks. (E) PD patients (4.9 ± 3.0) scored significantly higher ($p < 0.0001$) than iRBD patients (0.3 ± 0.8) and healthy controls (0.3 ± 0.7) on the Screening Questionnaire for Parkinsonism (SQP). (F) The RBD Screening Questionnaire (RBDSQ) revealed significant differences ($p < 0.0001$) between iRBD (9.0 ± 2.7) and PD patients (4.9 ± 3.0) or healthy controls (2.1 ± 2.2), and between PD patients and healthy controls. Significance was determined using GraphPad Prism Version 10.4.0 and the Kruskal-Wallis test (ns = non-significant, *$p < 0.05$, **$p < 0.01$, ****$p < 0.0001$).

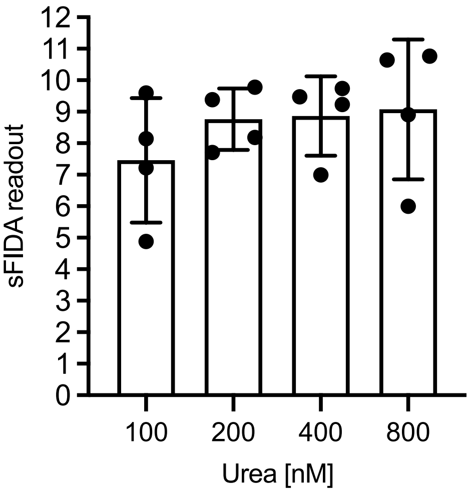

Figure S2: Urea concentrations between 100 and 800 mM do not affect the sFIDA readout. We prepared synthetic α-synuclein fibrils at a concentration equivalent to 8 pM of α-synuclein monomer, and measured them using sFIDA in the presence of increasing urea concentrations. Statistical analysis was performed using GraphPad Prism (Version 10.4.0) and the Kruskal-Wallis test, which revealed no significant differences between the measured values across urea concentrations.

Table S1. Demographic and clinical information and concentrations of α-synuclein aggregates in urine

Sample	Diagnosis	Sex	Age at sampling [yrs]	Age at onset [yrs]	Disease duration [yrs]	Education [yrs]	CCCSS score	DemTect score	NMSS score	MDS-UDPRS III score	Olfactory testing score	Hoehn and Yahr score	Levodopa challenge test % change	DaTSCAN score	Screening questionnaire PD score	RBDSQ score	α-Synuclein aggregate concentration [fM]
1	PD	Male	73	60	13	18	9	13	37	22	4	4	69	+	5	7	7.32
2	PD	Male	68	54	14	10	10	13	17	27	6	3	33	N/A	6	10	3.02
3	PD	Male	56	45	11	12	3	17	51	35	7	4	53	+	5	8	4.41
4	PD	Male	71	61	10	17	1	11	67	65	8	5	24	+	9	6	2.44
5	PD	Male	46	41	5	12	0	14	103	21	3	3	44	N/A	6	2	4.79
6	PD	Male	72	57	15	19	6	14	54	28	5	5	47	N/A	4	5	4.03
7	PD	Male	54	50	4	16	0	18	20	19	2	2	47	+	7	0	5.12
8	PD	Female	71	49	22	12	4	9	67	20	9	3	48	N/A	9	4	1.82
9	PD	Male	56	52	4	16	2	15	9	33	7	5	32	N/A	7	5	4.67
10	PD	Male	62	50	12	20	0	18	9	10	2	2	46	+	8	3	6.30
11	PD	Male	80	78	2	8	3	14	4	17	3	2	-	N/A	1	0	6.14
12	PD	Female	78	63	15	11	18	17	48	76	2	5	43	N/A	6	4	5.90
13	PD	Male	68	58	10	18	9	16	4	17	3	4	63	N/A	5	2	4.94
14	PD	Female	80	66	14	13	11	13	34	25	7	4	53	+	7	1	2.41
15	PD	Female	52	35	17	19	1	12	27	23	4	4	58	N/A	5	7	3.76
16	PD	Female	74	60	14	17	9	10	21	50	2	5	32	N/A	9	6	2.22
17	PD	Male	63	54	9	13	1	12	12	15	4	2	64	+	7	4	5.14
18	PD	Male	74	62	12	12	3	11	21	8	0	2	32	N/A	2	3	1.32
19	PD	Male	82	73	9	12	0	9	46	23	8	4	38	N/A	7	5	1.08
20	PD	Female	50	39	11	14	2	11	16	22	7	2	46	+	5	2	1.13
21	PD	Female	56	51	5	12	0	15	4	30	9	3	32	+	7	5	3.89
22	PD	Female	60	51	9	17	3	17	16	16	7	2	42	+	7	2	0.66

23	PD	Male	60	40	20	12	7	18	15	30	3	3	47	N/A	5	3	2.26
24	PD	Male	57	39	18	15	7	12	42	41	6	3	40	+	5	8	2.12
25	PD	Male	59	56	3	17	13	18	46	7	4	2	48	N/A	7	9	1.19
26	PD	Female	58	58	0	20	2	18	52	16	1	2	-	+	0	11	0.95
27	PD	Male	68	62	6	20	1	17	10	5	6	2	-	+	4	2	1.10
28	PD	Male	76	74	2	13	1	11	21	35	6	3	-	N/A	6	8	0.98
29	PD	Male	60	51	9	21	4	14	56	22	1	3	49	N/A	8	5	1.35
30	PD	Male	59	54	5	19	4	18	28	33	6	3	-	+	7	12	1.36
31	PD	Male	65	63	2	15	6	12	28	42	5	4	43	N/A	9	8	0.70
32	PD	Male	41	40	1	22	2	18	7	11	10	2	-	+	2	3	2.43
33	PD	Male	66	45	21	15	5	18	14	11	6	2	0	+	4	4	1.50
34	PD	Female	60	50	10	13	12	13	27	19	6	3	43	+	8	7	1.54
35	PD	Male	71	66	5	12	2	8	30	42	9	4	25	+	4	1	0.82
36	PD	Male	58	54	4	15	2	11	42	10	8	3	69	N/A	7	6	1.30
37	PD	Female	70	57	13	8	11	11	25	22	8	3	44	N/A	6	4	1.40
38	PD	Male	55	41	14	9	12	7	30	50	6	4	45	N/A	8	6	0.92
39	PD	Male	68	64	4	13	6	9	65	21	3	3	-	+	9	6	2.11
40	PD	Male	78	71	7	18	7	10	10	19	7	3	-	+	8	12	0.62
41	PD	Male	55	50	5	16	1	14	1	5	8	2	54	N/A	3	5	1.64
42	PD	Female	49	44	5	15	5	18	13	9	8	3	0	N/A	7	3	1.15
43	PD	Female	65	50	15	25	4	17	22	-	5	4	22	N/A	5	11	0.68
44	PD	Male	41	37	4	20	3	14	17	18	6	3	-	+	6	1	11.36
45	PD	Male	69	58	11	16	3	10	20	21	6	3	52	N/A	5	5	0.95
46	PD	Male	63	53	10	13	8	14	21	13	10	2	31	+	7	8	0.39
47	PD	Male	71	65	6	12	3	10	26	48	4	3	16	+	7	1	2.16
48	PD	Female	78	64	14	20	6	18	23	30	4	3	29	N/A	7	2	0.80
49	PD	Male	76	60	16	12	11	13	90	31	6	4	30	N/A	6	9	1.15
50	PD	Male	55	55	0	30	0	17	2	5	7	2	-	+	1	3	1.26
51	PD	Male	77	65	12	18	3	13	26	26	7	3	-2	N/A	4	8	0.54
52	PD	Male	64	52	12	22	9	16	20	25	7	2	93	N/A	8	4	1.86

53	PD	Male	83	73	10	22	8	18	36	54	5	4	-	N/A	6	7	1.29
54	PD	Male	62	53	9	18	3	15	44	6	2	2	33	+	5	6	1.33
55	PD	Female	70	63	7	14	0	12	18	13	6	3	37	N/A	8	4	1.73
56	PD	Female	74	61	13	8	3	12	19	63	4	5	-	N/A	7	7	1.28
57	PD	Female	77	50	27	13	1	18	67	30	2	4	57	N/A	9	7	1.10
58	PD	Female	62	48	14	23	7	18	11	40	7	5	9	+	6	4	16.76
59	PD	Female	75	49	26	16	2	9	36	25	2	3	-	N/A	5	4	0.98
60	PD	Male	59	57	2	14	1	14	29	41	9	3	-	N/A	4	3	2.31
61	PD	Male	68	58	10	14	0	18	24	24	0	2	61	N/A	7	4	1.46
62	PD	Male	73	68	5	18	0	18	14	22	7	3	-	+	3	4	1.18
63	PD	Male	57	49	8	15	1	14	13	10	9	2	55	N/A	3	2	3.38
64	PD	Female	54	45	9	16	2	18	15	9	10	3	69	+	7	3	0.65
65	PD	Female	57	45	12	12	2	18	122	26	7	2	72	N/A	8	7	0.98
66	PD	Female	68	51	17	12	0	17	19	19	8	4	32	N/A	2	8	1.18
67	PD	Female	64	57	7	20	8	13	59	21	8	3	63	N/A	8	11	0.69
68	PD	Male	56	48	8	13	10	12	39	22	4	3	31	+	6	6	0.77
69	PD	Female	74	73	1	17	2	15	15	16	8	3	-	+	6	9	1.12
70	PD	Male	46	36	10	24	1	15	22	16	9	2	67	+	6	3	1.06
71	PD	Female	69	56	13	11	7	15	51	28	4	3	42	N/A	7	6	0.59
72	PD	Male	55	53	2	18	12	14	50	17	3	2	-	N/A	8	6	2.26
73	PD	Male	78	60	18	17	2	12	36	59	3	5	-	N/A	7	5	2.02
74	PD	Male	52	45	7	16	7	9	51	21	8	3	35	+	6	2	0.75
75	PD	Female	57	55	2	15	2	18	12	6	6	3	73	+	1	1	0.69
76	PD	Male	55	45	10	-	-	7	79	22	7	3	48	+	-	-	90.28
77	PD	Male	60	57	3	10	0	12	55	36	2	3	-	+	1	1	1.01
78	PD	Male	52	46	6	18	6	13	0	5	6	2	0	N/A	3	1	1.18
79	PD	Male	51	40	11	14	7	8	10	31	4	4	49	+	5	5	0.69
80	PD	Male	73	72	1	14	15	15	62	23	1	3	-	N/A	5	1	0.64
81	PD	Male	66	55	11	12	0	14	51	13	7	2	-	+	4	2	0.92
82	PD	Female	67	53	14	12	3	15	57	31	8	4	34	N/A	9	10	0.90

83	PD	Male	72	66	6	13	1	15	13	12	5	3	-	N/A	7	9	1.20
84	PD	Male	59	49	10	13	3	14	29	13	3	2	47	N/A	7	2	0.92
85	PD	Male	74	68	6	8	2	9	6	42	6	-	54	N/A	4	1	1.83
86	PD	Female	62	56	6	16	0	13	13	10	2	2	-	N/A	2	6.	2.06
87	PD	Male	71	64	7	19	0	18	18	29	3	3	-	N/A	5	1	2.07
88	PD	Male	51	51	0	13	1	17	12	11	3	2	-	+	4	11	1.40
89	PD	Female	57	56	1	14	3	13	24	6	8	2	-	+	5	4	1.71
90	PD	Male	78	72	6	13	0	17	11	14	6	3	44	+	2	1	0.91
91	PD	Male	62	59	3	22	1	14	13	7	7	2	-	+	2	3	2.08
92	PD	Male	64	63	1	18	0	13	12	4	2	2	-	+	3	3	1.50
93	PD	Male	80	77	3	10	1	13	31	17	1	2	14	+	4	3	2.28
94	iRBD	Male	56	-	-	21	2	17	6	1	11	-	-	N/A	0	6	1.02
95	iRBD	Male	72	60	12	3	-	16	1	4	11	-	-	N/A	2	6	1.33
96	iRBD	Male	61	-	-	16	-	14	4	5	11	-	-	N/A	0	6	1.47
97	iRBD	Female	59	55	4	20	4	17	14	0	6	-	-	N/A	0	10	1.42
98	iRBD	Male	78	-	-	14	-	17	15	4	8	-	-	N/A	0	10	1.13
99	iRBD	Female	67	50	17	12	3	13	-	8	5	-	-	N/A	0	9	2.40
100	iRBD	Male	63	63	0	16	2	17	3	5	10	-	-	N/A	0	8	0.64
101	iRBD	Male	58	54	4	16	0	13	-	6	9	-	-	N/A	0	10	0.98
102	iRBD	Female	64	61	3	21	0	15	-	3	3	-	-	N/A	0	7	1.82
103	iRBD	Male	64	63	1	15	4	11	-	2	7	-	-	+	0	8	1.35
104	iRBD	Male	70	60	10	-	0	14	12	5	10	-	-	N/A	0	12	8.19
105	iRBD	Male	66	52	14	17	0	12	4	5	7	-	-	+	0	11	1.25
106	iRBD	Male	73	71	2	-	-	12	2	4	6	-	-	N/A	-	-	0.47
107	iRBD	Female	65	60	5	16	1	17	6	3	5	-	-	+	0	9	0.80
108	iRBD	Male	72	62	10	11	2	14	-	5	9	-	-	+	0	-	1.30
109	iRBD	Male	75	72	3	18	2	14	8	7	3	-	-	N/A	0	7	0.35
110	iRBD	Male	69	64	5	14	0	17	9	4	8	-	-	+	0	12	0.87
111	iRBD	Female	66	62	4	15	5	18	2	3	9	-	-	N/A	0	5	3.82
112	iRBD	Male	59	56	3	19	3	9	22	5	7	-	-	+	0	9	1.04

113	iRBD	Male	75	66	9	18	0	17	1	5	7	-	-	N/A	0	11	1.42
114	iRBD	Male	55	40	15	21	0	11	0	8	12	-	-	N/A	0	5	1.27
115	iRBD	Male	69	66	3	15	8	12	-	13	7	-	-	+	0	11	1.85
116	iRBD	Male	54	49	5	12	5	12	48	3	8	-	-	+	5	13	0.90
117	iRBD	Male	74	72	2	16	0	15	-	6	7	-	-	N/A	0	10	1.03
118	iRBD	Male	66	61	5	15	3	18	21	7	6	-	-	N/A	0	10	1.01
119	iRBD	Male	70	63	7	20	2	13	31	8	1	-	-	N/A	1	5	2.93
120	iRBD	Male	67	50	17	13	0	17	2	10	7	-	-	N/A	0	10	1.35
121	iRBD	Male	67	56	11	17	0	17	3	4	9	-	-	+	0	10	4.52
122	iRBD	Male	62	47	15	31	4	14	-	5	9	-	-	+	1	9	2.37
123	iRBD	Male	70	-	-	15	0	18	1	4	7	-	-	N/A	0	5	1.03
124	iRBD	Female	63	62	1	19	12	14	-	0	4	-	-	N/A	0	8	0.63
125	iRBD	Male	75	55	20	15	6	13	-	4	8	-	-	+	1	8	1.42
126	iRBD	Male	67	61	6	16	5	13	23	1	3	-	-	N/A	0	11	2.82
127	iRBD	Male	70	67	3	22	3	14	-	3	3	-	-	+	1	10	2.06
128	iRBD	Male	74	72	2	12	2	11	-	5	5	-	-	N/A	0	7	1.90
129	iRBD	Male	58	53	5	13	9	14	-	7	5	-	-	+	3	11	1.03
130	iRBD	Female	77	66	11	16	1	15	4	5	10	-	-	N/A	0	12	1.01
131	iRBD	Female	59	55	4	16	1	15	-	4	10	-	-	N/A	0	7	1.03
132	iRBD	Male	68	60	8	18	2	18	-	4	7	-	-	N/A	0	9	0.80
133	iRBD	Male	63	-	-	-	-	18	-	2	7	-	-	N/A	-	-	1.74
134	iRBD	Male	64	62	2	12	5	14	102	5	10	-	-	N/A	0	13	1.19
135	iRBD	Male	61	50	11	16	6	18	76	5	9	-	-	+	0	12	1.88
136	iRBD	Male	64	60	4	-	-	11	9	2	5	-	-	N/A	-	-	0.73
137	iRBD	Male	66	63	3	12	3	13	2	5	7	-	-	N/A	0	4	2.33
138	iRBD	Male	63	62	1	15	2	14	9	0	3	-	-	N/A	0	5	2.75
139	iRBD	Male	61	51	10.	25	1	15	6	3	5	-	-	+	1	12	1.99
140	iRBD	Male	58	54	4	15	2	17	6	1	6	-	-	+	0	3	1.15
141	iRBD	Male	63	55	8	13	9	11	8	3	0	-	-	N/A	0	11	1.95
142	iRBD	Male	59	47	12	22	2	14	5	2	9	-	-	N/A	0	11	1.66

143	iRBD	Male	73	63	10	17	0	12	1	1	8	-	-	N/A	0	8	3.43
144	iRBD	Male	69	54	15	18	8	18	14	0	6	-	-	+	0	10	3.03
145	iRBD	Male	60	-	-	-	-	12	15	3	7	-	-	N/A	-	-	3.95
146	iRBD	Male	63	53	10	17	0	18	25	3	2	-	-	N/A	0	7	3.27
147	iRBD	Male	60	50	10	15	2	17	7	2	7	-	-	+	0	2	2.69
148	iRBD	Male	74	71	3	19	1	18	18	9	9	-	-	+	0	12	4.28
149	iRBD	Male	56	53	3	-	-	14	17	2	10	-	-	N/A	-	-	2.51
150	iRBD	Male	72	58	14	8	1	17	12	1	5	-	-	N/A	0	12	3.41
151	iRBD	Male	65	61	4	13	1	18	6	3	4	-	-	N/A	0	7	0.84
152	iRBD	Male	74	68	6	12	6	15	13	2	6	-	-	N/A	0	10	1.39
153	iRBD	Male	72	57	15	17	0	14	19	5	5	-	-	N/A	0	10	0.52
154	iRBD	Male	67	65	2	11	4	12	11	1	3	-	-	+	0	6	1.71
155	iRBD	Male	80	60	20	19	0	14	15	7	6	-	-	N/A	0	7	0.94
156	iRBD	Male	68	63	5	18	6	15	40	2	4	-	-	+	0	12	1.17
157	iRBD	Female	77	70	7	13	6	14	24	4	6	-	-	+	1	7	1.45
158	iRBD	Male	74	69	5	17	1	14	16	2	0	-	-	N/A	0	11	1.21
159	iRBD	Male	69	64	5	13	4	15	21	7	4	-	-	N/A	0	10	0.66
160	iRBD	Male	60	59	1	11	3	16	29	1	4	-	-	N/A	1	13	0.89
161	iRBD	Male	61	59	2	-	-	18	15	2	8	-	-	N/A	-	-	1.07
162	iRBD	Male	57	38	19	17	0	15	38	3	7	-	-	N/A	0	12	0.57
163	iRBD	Female	56	50	6	13	4	17	27	2	9	-	-	N/A	1	12	1.55
164	iRBD	Male	68	48	20	-	-	11	40	-	7	-	-	N/A	-	-	1.66
165	iRBD	Male	76	70	6	15	3	15	18	12	10	-	-	N/A	1	11	3.78
166	HC	Male	21	-	-	15	-	15	-	-	-	-	-	N/A	0	7	1.04
167	HC	Male	18	-	-	12	3	15	-	-	10	-	-	N/A	0	2	1.84
168	HC	Male	70	-	-	16	7	10	-	-	11	-	-	N/A	0	0	2.15
169	HC	Female	58	-	-	16	0	17	-	-	11	-	-	N/A	0	0	0.72
170	HC	Male	70	-	-	12	0	12	-	-	10	-	-	N/A	0	1	2.68
171	HC	Female	55	-	-	15	1	18	-	-	11	-	-	N/A	0	1	1.34
172	HC	Female	20	-	-	14	4	15	-	-	11	-	-	N/A	0	1	0.82

173	HC	Female	20	-	-	14	5	16	-	-	10	-	-	N/A	0	3	0.60
174	HC	Female	78	-	-	12	4	18	-	-	11	-	-	N/A	0	3	1.16
175	HC	Female	66	-	-	14	0	17	-	-	11	-	-	N/A	0	3	2.48
176	HC	Male	75	-	-	18	0	14	27	-	11	-	-	N/A	0	0	0.72
177	HC	Male	74	-	-	10	2	13	8	-	10	-	-	N/A	0	0	1.48
178	HC	Female	70	-	-	11	0	18	9	-	11	-	-	N/A	3	2	1.11
179	HC	Female	63	-	-	20	4	18	1	-	11	-	-	N/A	0	2	0.50
180	HC	Female	66	-	-	18	7	18	10	-	12	-	-	N/A	0	2	0.38
181	HC	Male	34	-	-	16	7	17	1	-	11	-	-	N/A	0	1	0.63
182	HC	Female	72	-	-	18	1	18	10	-	10	-	-	N/A	1	0	0.71
183	HC	Male	77	-	-	23	5	15	0	-	11	-	-	N/A	0	2	0.42
184	HC	Female	60	-	-	16	0	18	18	-	12	-	-	N/A	0	4	1.33
185	HC	Female	49	-	-	12	-	17	-	-	-	-	-	N/A	0	6	0.52
186	HC	Female	64	-	-	13	2	18	-	-	-	-	-	N/A	1	2	0.79
187	HC	Female	61	-	-	14	2	17	3	-	8	-	-	N/A	0	1	0.71
188	HC	Male	36	-	-	20	0	18	4	-	11	-	-	N/A	0	1	1.96
189	HC	Male	62	-	-	13	1	-	-	-	-	-	-	N/A	2	2	0.57
190	HC	Female	60	-	-	20	0	18	18	-	2	-	-	N/A	0	0	0.55
191	HC	Male	52	-	-	15	0	14	4	-	10	-	-	N/A	0	0	1.96
192	HC	Female	66	-	-	19	2	15	1	-	10	-	-	N/A	0	0	0.68
193	HC	Female	63	-	-	12	4	17	34	-	11	-	-	N/A	0	0	0.77
194	HC	Male	59	-	-	16	1	-	11	-	11	-	-	N/A	0	2	0.63
195	HC	Female	71	-	-	16	11	18	15	-	11	-	-	N/A	0	4	0.48
196	HC	Female	70	-	-	12	0	17	16	-	6	-	-	N/A	0	4	0.72
197	HC	Female	48	-	-	17	1	18	1	-	11	-	-	N/A	0	0	0.58
198	HC	Male	65	-	-	18	2	18	4	-	11	-	-	N/A	0	2	0.28
199	HC	Male	72	-	-	22	1	13	14	-	7	-	-	N/A	0	3	0.49
200	HC	Female	50	-	-	15	2	18	18	-	11	-	-	N/A	0	5	0.44
201	HC	Male	62	-	-	16	-	13	-	-	-	-	-	N/A	0	9	0.70
202	HC	Male	43	-	-	21	0	14	0	-	10	-	-	N/A	0	0	1.02

203	HC	Male	57	-	-	22	0	15	3	-	11	-	-	N/A	0	1	1.25
204	HC	Female	69	-	-	21	0	16	2	-	7	-	-	N/A	0	0	0.42
205	HC	Female	48	-	-	13	3	17	3	-	12	-	-	N/A	0	0	0.48
206	HC	Female	39	-	-	18	4	15	3	-	11	-	-	N/A	0	0	0.73
207	HC	Female	42	-	-	17	3	18	21	-	11	-	-	N/A	0	0	0.85
208	HC	Female	26	-	-	18	-	18	-	-	-	-	-	N/A	0	7	0.94
209	HC	Female	53	-	-	15	1	17	2	-	11	-	-	N/A	0	3	0.46
210	HC	Female	24	-	-	18	6	17	18	-	11	-	-	N/A	2	1	1.60
211	HC	Female	52	-	-	15	4	13	46	-	11	-	-	N/A	0	4	1.24
212	HC	Male	78	-	-	12	0	13	32	-	-	-	-	N/A	0	5	2.43
213	HC	Female	54	-	-	13	-	16	-	-	-	-	-	N/A	3	7	0.38
214	HC	Male	26	-	-	13	0	18	2	-	11	-	-	N/A	0	0	0.45
215	HC	Male	38	-	-	17	3	17	0	-	12	-	-	N/A	0	3	0.41
216	HC	Female	61	-	-	23	5	18	16	-	9	-	-	N/A	1	1	0.72
217	HC	Male	64	-	-	18	3	13	20	-	11	-	-	N/A	0	2	0.64

PD = Parkinson’s disease; iRBD = isolated rapid eye movement sleep behavior disorder; HC = healthy control; CCCSS = Cleveland Clinic Constipation Scoring System; NMSS = Non-Motor Symptoms Scale; MDS-UPDRS III = Movement Disorder Society’s Unified Parkinson's Disease Rating Scale Part III; RBDSQ = iRBD screening questionnaire; N/A = not available.

Table S2. The coefficient of variation of each SiNaP concentration

Concentration [fM]	Coefficient of variation [%]
0.1	18.2
0.32	31.7
1	44.0
3.2	35.3
10	7.9
32	16.5
100	13.8
320	12.0
1000	10.7
3200	9.0
Mean	19.9

Table S3. The Spearman coefficient of correlation reveals no significant correlation between the α-synuclein aggregate concentrations in urine and other disease-relevant scores

	PD	iRBD	HC
Age	–0.089	0.059	–0,010
Education	0.028	0.025	–0.253
Sex	–0.166	–0.029	–0.135
DemTect	0.025	–0.033	–0.279
Disease duration	0.019	0.212	N/A
MDS-UPDRS III	0.079	–0.098	N/A
Screening questionnaire for parkinsonism	–0.032	0.094	0.0003
RBDSQ	–0.171	–0.050	–0.119

PD = Parkinson’s disease; iRBD = isolated rapid eye movement sleep behavior disorder; HC = healthy control; MDS-UPDRS III = Movement Disorder Society’s Unified Parkinson's Disease Rating Scale Part III; N/A = not applicable; RBDSQ = REM sleep behavior disorder screening questionnaire. The Spearman coefficient of correlation was determined with GraphPad Prism Version 10.4.0.
